# Bioconversion of Agricultural Residues into Nutrient-Enriched Biofertilizers Using *Trichoderma* sp.: Effects of Substrate Type and Incubation Time

**DOI:** 10.3390/biology15191712

**Published:** 2026-09-26

**Authors:** Oluwaseyi Matthew Abioye, Oluwaseun Temitope Faloye, Kamorudeen Olaniyi Yusuf, Praise Iyeke, Laemthong Laokhongthavorn, Peter Pelumi Ikubanni, Viroon Kamchoom

**Affiliations:** 1 Department of Agricultural and Bio-Systems Engineering, Landmark University, Omu-Aran PMB 1001, Kwara State, Nigeria; 2 Department of Water Resources Management and Agrometeorology, Federal University, Oye-Ekiti PMB 373, Ekiti State, Nigeria; oluwaseun.faloye@fuoye.edu.ng; 3 Department of Civil Engineering, Landmark University, Omu-Aran PMB 1001, Kwara State, Nigeria; 4 Department of Agricultural and Bio-Systems Engineering, University of Ilorin, Ilorin PMB 1515, Kwara State, Nigeria; 5 Excellent Centre for Green and Sustainable Infrastructure, School of Engineering, King Mongkut’s Institute of Technology Ladkrabang, Bangkok 10520, Thailand; 6 Department of Mechatronics Engineering, Bowen University, Iwo PMB 23401, Osun State, Nigeria

**Keywords:** *Trichoderma* sp., agricultural waste, valorization, biofertilizer production, nutrient mineralization, circular bioeconomy

## Abstract

Improper disposal or open burning of agricultural waste releases smoke, particulate matter, and greenhouse gases, contributing to air pollution and climate change. The utilization of agricultural wastes as substrates for biofertilizer production offers a sustainable approach to waste valorization and nutrient recycling. This study evaluated the effects of agricultural waste type and contact time on the nutrient composition of *Trichoderma* sp.-based biofertilizer produced from sawdust, rice husk, and guinea corn husk. A factorial experiment was conducted using four substrates and four incubation periods (0, 5, 10, and 15 days). Nutrient parameters, including total nitrogen (TN %), phosphorus (P %), and potassium (K %), were determined and subjected to analysis of variance. The biofertilizer exhibited high microbial viability, with a highest propagule density of 6.46 × 10^11^ CFU ml^−1^, indicating successful fungal colonization. Significant (*p* < 0.0001) effects of substrate type, contact time, and their interaction were observed for all nutrient parameter accumulation. Total nitrogen increased with incubation time, reaching a maximum of 0.901% in guinea corn husk after 15 days. Rice husk recorded the highest phosphorus concentration (2.497%) at 15 days, while sawdust produced the greatest potassium concentration (2.389%) at 15 days. The results indicate that *Trichoderma* sp. effectively bio-converts agricultural residues into nutrient-rich biofertilizers, with substrate type influencing nutrient profiles. This technology represents a promising strategy for sustainable soil fertility management and circular agricultural production systems, depending on substrate type used and incubation time.

## Simple Summary

The study demonstrated the potential of *Trichoderma* sp. to convert agricultural wastes into nutrient-enriched biofertilizers. Significant effects of substrate type and incubation period were observed on nitrogen, phosphorus, and potassium concentrations. Guinea corn husk yielded the highest nitrogen content (0.901%), rice husk recorded the greatest phosphorus concentration (2.497%), while sawdust yielded the highest potassium content (2.389%) after 15 days of incubation. Nutrient accumulation generally increased with contact time, reflecting enhanced microbial decomposition and mineralization of organic substrates. The findings highlight the feasibility of utilizing locally available agricultural residues for sustainable biofertilizer production, nutrient recycling, and environmentally friendly soil fertility management.

## 1. Introduction

The increasing global demand for agricultural productivity has led to the extensive use of synthetic fertilizers to enhance crop yields. Synthetic nitrogen fertilizer produced via the Haber–Bosch process is credited with enabling food production for roughly half of humanity [1], while inorganic fertilizers have contributed significantly to global food production. However, their excessive and prolonged use has resulted in numerous environmental challenges, including soil degradation, nutrient imbalance, groundwater contamination, greenhouse gas emissions, and declining soil microbial diversity [2,3]. These concerns have intensified the search for environmentally friendly and sustainable alternatives capable of maintaining soil fertility while reducing dependence on chemical inputs. Biofertilizers represent one such alternative because of their environmental benefits, particularly their potential to reduce groundwater contamination. Moreover, they can complement synthetic and inorganic fertilizers, thereby reducing dependence on chemical inputs while sustaining or improving agricultural productivity.

Biofertilizers have emerged as promising tools for sustainable agriculture because of their ability to improve nutrient availability, stimulate plant growth, and enhance soil biological activity. Unlike chemical fertilizers, biofertilizers utilize beneficial microorganisms to facilitate nutrient cycling, organic matter decomposition, and the mobilization of essential plant nutrients [4]. Among the diverse microbial agents used in biofertilizer production, species of the genus *Trichoderma* have attracted considerable attention because of their multifunctional roles in agriculture. In addition to their well-documented biocontrol properties, *Trichoderma* sp. contribute to nutrient mineralization, phosphorus solubilization, organic matter degradation, and the promotion of plant growth through the production of phytohormones and other bioactive compounds [5,6]. *Trichoderma* sp. is particularly recognized for its strong enzymatic capabilities, including the production of cellulases, hemicellulases, proteases, and lignin-degrading enzymes that facilitate the decomposition of organic materials [7]. These characteristics make *Trichoderma* sp. an ideal candidate for converting agricultural residues into nutrient-rich biofertilizers. The fungal degradation of lignocellulosic biomass not only enhances nutrient release but also contributes to waste reduction and resource recovery, thereby supporting the principles of a circular bioeconomy [8].

Agricultural activities generate substantial quantities of crop residues worldwide, including rice husks, sawdust, maize residues, and sorghum husks. A significant proportion of these residues is either burned in the field or disposed of improperly, resulting in environmental pollution and the loss of potentially valuable organic resources [9]. The conversion of such agricultural wastes into biofertilizers represents an effective strategy for waste valorization while simultaneously improving soil fertility and agricultural sustainability. Agricultural residues contain varying amounts of cellulose, hemicellulose, lignin, and mineral nutrients, which influence their suitability as substrates for microbial growth and nutrient transformation [10]. Consequently, the type of agricultural waste used may significantly affect the quality and nutrient composition of the resulting biofertilizer.

The efficiency of microbial biofertilizer production is also influenced by incubation (contact time). During fermentation, microbial colonization and enzymatic degradation progressively transform complex organic compounds into simpler forms, thereby enhancing nutrient availability [11]. The duration of incubation can therefore determine the extent of substrate decomposition, microbial biomass accumulation, and nutrient enrichment. However, optimal incubation periods may vary depending on the physicochemical characteristics of the substrate and the metabolic activity of the microbial inoculant. Although several studies have reported the potential of *Trichoderma* sp. in composting and organic waste bioconversion, comparative information on the influence of different agricultural substrates and incubation periods remains limited. For example, Gary E. Harman et al. [5] reported that *Trichoderma* spp. possess strong cellulolytic and ligninolytic capabilities that facilitate the decomposition of organic residues and enhance nutrient availability. Similarly, Irina S. Druzhinina et al. [7] highlighted the extensive enzymatic machinery of *Trichoderma* spp., including cellulases, hemicellulases, and chitinases, which contribute to the degradation of lignocellulosic biomass. Singh et al. [12] observed improved nutrient mineralization and compost quality following inoculation of agricultural residues with *Trichoderma harzianum*. In a study on crop residue management, Elrys et al. [13] found that *Trichoderma*-assisted decomposition accelerated organic matter breakdown and increased nitrogen and phosphorus availability. Likewise, Asghar et al. [14] reported that *Trichoderma* spp. significantly enhanced biomass degradation, nutrient recycling, and soil fertility through the conversion of lignocellulosic wastes into value-added biofertilizers. Collectively, these findings demonstrate the considerable potential of *Trichoderma* sp. as biological agents for sustainable waste valorization and nutrient recovery.

However, despite the large number of studies on the use of *Trichoderma* as a biofertilizer, limited information is available regarding the comparative suitability of different agricultural wastes for *Trichoderma* sp.-based biofertilizer production and the influence of contact time on nutrient enrichment. In addition, the interaction between agricultural waste type and contact time has been scarcely investigated. Understanding these interactions is essential for optimizing biofertilizer production processes and maximizing nutrient recovery from agricultural residues. Therefore, this study evaluated the effects of three agricultural waste substrates—sawdust, rice husk, and guinea corn husk—and different contact times on the nutrient composition of *Trichoderma* sp. (presumably *Trichoderma viride*-based biofertilizer). Specifically, the study assessed variations in total nitrogen, phosphorus, and potassium concentrations and examined the main interaction between substrate type and incubation period. We hypothesized that biofertilizer concentration is dependent on substrate type and incubation time. The findings are expected to contribute to the development of sustainable biofertilizer production technologies that promote agricultural waste valorization, nutrient recycling, and environmentally responsible soil fertility management.

## 2. Materials and Methods

### 2.1. Study Materials

Agricultural waste materials comprising sawdust, rice husk, and guinea corn husk were collected from local processing facilities and farms in Omu Aran, Kwara State, Nigeria, in May, 2024. About 1 kg of each substrate was collected in the month of May, 2024. The substrates were selected because of their abundance, biodegradability, and potential to support fungal growth. All chemicals and reagents used for nutrient analyses were of analytical grade. The fungus was purified through repeated sub-culturing on Potato Dextrose Agar (PDA) and identified based on its morphological characteristics and microscopic features following standard mycological procedures [15,16]. The microscopic approach has been similarly used in some studies for fungal identification [17]. Pure cultures were subsequently maintained on PDA slants at 4 °C until use. Potato Dextrose Agar (PDA) was prepared according to the manufacturer’s instructions (PDA; HiMedia Laboratories Pvt. Ltd., Mumbai, India), consisting of potato infusion (200 g L^−1^), dextrose (20 g L^−1^), and agar (15 g L^−1^), and was used for culturing *Trichoderma* sp.

### 2.2. Preparation of Agricultural Waste Substrates

The collected agricultural wastes were cleaned manually to remove foreign materials and debris. The substrates were air-dried and milled to obtain a uniform particle size (≤2 mm) suitable for fungal colonization. Each substrate was sterilized to eliminate contaminating microorganisms before inoculation. Sterilization was carried out in an autoclave (TEMPO laboratory autoclave (Series TI-121, TEMPO Instruments, Mehler Bayandal, India) at 121 °C and 15 psi for 15–20 min following standard microbiological procedures [18].

#### Preparation of *Trichoderma* Species Inoculum

Pure cultures of *Trichoderma* sp. were sub-cultured on Potato Dextrose Agar and incubated at 28 ± 2 °C for 5 days until sufficient sporulation occurred. Spore suspensions were prepared by flooding the culture plates with sterile distilled water and gently scraping the colony surface. The concentration of spores was adjusted to obtain a uniform inoculum. The viability of the fungal inoculum was determined by serial dilution and colony-forming unit (CFU) enumeration according to standard microbiological methods [19].

Freshly decomposed bamboo litter was collected aseptically from a bamboo plantation within the University of Ilorin, Nigeria, and transported to the laboratory in sterile polyethylene bags. Ten grams (10 g) of the decomposed bamboo litter were transferred into a sterile 1000 mL Erlenmeyer flask containing 90 mL of sterile physiological saline solution (0.85% NaCl). The suspension was shaken vigorously on an orbital shaker at approximately 150 rpm for 20–30 min to detach fungal propagules from the litter particles, producing a 10^−1^ dilution.

Serial tenfold dilutions were prepared by transferring 1 mL of the suspension into 9 mL of sterile diluent to obtain successive dilutions (10^−4^–10^−9^). Using the spread-plate technique, 0.1 mL aliquots from appropriate dilutions (10^−4^–10^−9^) were aseptically inoculated onto Potato Dextrose Agar (PDA) plates supplemented with chloramphenicol (50 mg L^−1^) to suppress bacterial growth. The PDA medium consisted of potato infusion (200 g L^−1^), dextrose (20 g L^−1^), and agar (15 g L^−1^). The combined morphological evidence (rapid growth, white-to-pale-yellow mycelium, pyramidal conidiophore branching, lageniform phialides, globose green conidia at maturity, and chlamydospore production) was used to diagnostically identify *Trichoderma* sp., and subsequently maintained as a pure culture in the culture collection of the Department of Forestry, University of Ilorin, from which it was retrieved for the present study, and assigned the strain code *T. viride* UIFF-BL01.

*Trichoderma* sp. inoculum was prepared and standardized to a propagule density of 6.46 × 10^11^ CFU ml^−1^, which was determined from nine independent replicates using Equation (1) below.

(1) $$\mathrm{CFU}/\mathrm{mL}=\frac{Number\;of\;colonies\;\times \;Dilution\;factor}{Volume\;plated}$$

This was determined by spread-plating on *Trichoderma*-Selective Medium (TSM). Plates were incubated for 5 days, and counts were recorded exclusively from dilutions yielding between 25 and 250 colony-forming units per plate, to guarantee reliable counts. Ten milliliters of the standardized inoculum was then applied per 250 g carrier substrate (guinea corn husk, sawdust and rice husk). Particle size of ≤2 mm was maintained for all substrates. Fermentation was conducted in 1000 mL Pyrex^®^ borosilicate glass Erlenmeyer flasks (Corning Incorporated, Corning, New York, NY, USA).

### 2.3. Biofertilizer Production

A factorial experiment consisting of three agricultural waste substrates (sawdust, rice husk, and guinea corn husk) and four contact times (0, 5, 10, and 15 days) was established. Equal quantities of each sterilized substrate were placed in sterile fermentation containers and inoculated with the prepared *Trichorderma* sp. spore suspension. The moisture content was maintained at approximately 60–70% on a wet basis throughout the fermentation process to support fungal growth [10]. The initial substrate moisture was approximately 70% and flasks were closed with cotton plugs for the exchange to minimize contamination and loss of water by evaporation. The moisture content was determined gravimetrically by weighing each flask at 24 h intervals, the moisture loss did not exceed 1% of the total flask mass during the incubation periods, and no additional water was added. Improved aeration was achieved by loosely covering the containers, while periodic manual mixing by spatula at intervals during incubation ensured uniform aeration and colonization of the fungi. The fermentation containers were completely randomized throughout the incubation period to avoid biases in the measurement.

The uninoculated and inoculated substrates were incubated at room temperature (28 ± 2 °C) under aerobic conditions. The uninoculated substrates were similarly sterilized and followed the same incubation conditions with the inoculated substrates. Samples were collected after 5, 10, and 15 days of incubation for nutrient analysis. Uninoculated substrates, namely sawdust, guinea corn husk and rice husk, served as controls for comparison. The sawdust was obtained from the sawmill at Omu Aran, Kwara State, Nigeria. The study region was located at latitude (8°8′24.72″ N) and longitude (5°5′46.68″ E). The site experiences an average annual temperature of 29.54 °C (85.17 °F), which is 0.08% warmer than the average for Nigeria. The sawdust was from rosewood (*Dalbergia latifolia*) which has been planted for more than 30 years, which is common within the locality. Also, guinea corn and rice husks were obtained from the marketers in Omu Aran in May, 2024. The sawdust was from rosewood of more than 30 years. The rice and guinea corn husk were still fresh when obtained from the local marketers. All substrates were allowed to air dry until constant weight was achieved.

As such, we used the uninoculated sawdust, guinea corn husk and rice husk as controls, and then compared the results of the N, P and K concentrations at 5, 10 and 15 days of contact time with the control.

### 2.4. Determination of Nutrient Composition

The inoculum nutrient concentrations of sodium, calcium, magnesium, manganese, and iron were determined after digestion. The inoculum liquid was thoroughly mixed so that fungal cells/spores and any suspended material were uniformly distributed. Thereafter, 10 mL of the inoculum was measured and transferred into a digestion vessel. For digestion to take place, nitric acid (HNO_3_) was added and allowed to remain for 30 min to ensure digestion had taken place. After digestion, the solution was allowed to cool and diluted with deionized water. Sodium concentration was determined using flame photometry, while other parameters were determined using the atomic absorption spectrometry (AAS).

Total nitrogen was determined using the Kjeldahl digestion method as described by AOAC [20]. Briefly, dried biofertilizer samples of approximately 0.5 g were digested with 10 mL of concentrated sulfuric acid in the presence of a catalyst mixture (containing potassium sulfate and copper sulfate). The digest was neutralized, distilled, and titrated to determine nitrogen concentration. Results were expressed as percentage nitrogen (% N).

Total phosphorus was determined using the colorimetric method following acid digestion of the biofertilizer samples. Approximately 0.5 g of oven-dried and finely ground biofertilizer sample was accurately weighed into a digestion tube. A digestion mixture consisting of 10 mL concentrated nitric acid (HNO_3_ (Merck, Darmstadt, Germany)) and 4 mL perchloric acid (HClO_4_) (Merck, Darmstadt, Germany) was added. After cooling, the digest was quantitatively transferred into a 100 mL volumetric flask and diluted to volume with deionized water. The solution was filtered through Whatman No. 42 filter paper (Whatman, Lagos, Nigeria) where necessary to remove any insoluble residue. An aliquot (5 mL) of the digested sample was transferred into a 50 mL volumetric flask, followed by the sequential addition of 5 mL ammonium molybdate–antimony potassium tartrate reagent (Merck, Darmstadt, Germany) and 8 mL freshly prepared ascorbic acid solution. The mixture was diluted to volume with distilled water, thoroughly mixed, and allowed to stand for 20–30 min at room temperature to permit development of the characteristic molybdenum blue color. The absorbance of the developed color was measured using a spectrophotometer at wavelength of 882 nm, and phosphorus concentration was calculated from a standard calibration curve. Potassium concentration was determined after wet digestion of the samples using a flame photometer at a wavelength of 766.5 nm, according to standard procedures described by AOAC [20]. Approximately 0.5 g of finely ground biofertilizer sample was accurately weighed into a 100 mL digestion tube. A digestion mixture consisting of 10 mL concentrated nitric acid (HNO_3_, 65%) (Merck, Darmstadt, Germany) and 4 mL perchloric acid (HClO_4_, 70%) (Merck, Darmstadt, Germany) was added to each sample. The tubes were left to stand for approximately 30 min to allow pre-digestion before heating. The samples were then digested on a digestion block at 180–200 °C until dense white fumes appeared and a clear, colorless solution was obtained, indicating complete oxidation of the organic matter. The digests were allowed to cool to room temperature and quantitatively transferred into 100 mL volumetric flasks, after which the volume was made up with deionized water. The digested solutions were filtered through Whatman No. 42 filter paper to remove any undigested particles. Potassium values were then expressed as percentage K. The same approach was used for the determination of the total phosphorus for the inoculum. Also, a similar approach as used for the determination of the substrate-biofertilizer total potassium was used for the inoculum. During the laboratory analysis, calibration curves were prepared using certified standard solutions and reagent blanks accompanied each digestion batch. All nutrient analyses were performed with nine replicates from independent containers and nutrient concentrations were expressed on a dry-weight basis following oven drying. Instrument calibration was verified before each analytical session.

Total carbon of the substrates was determined using the Walkley–Black wet oxidation method. The substrate samples (sawdust, guinea corn and rice husks) were oven-dried, ground and passed through a sieve. A mass of 0.5 g of the sample was weighed into a digestion flask. A measured volume of potassium dichromate (K_2_Cr_2_O_7_) (Merck, Darmstadt, Germany) solution was then added. Concentrated sulfuric acid (H_2_SO_4_) (Merck, Darmstadt, Germany) was added to oxidize the organic carbon. The mixture was then allowed to digest for about 30 min. The excess dichromate was thereafter titrated with ferrous ammonium sulfate (FAS) using an indicator. A reagent blank was also analyzed alongside the samples. The percentage organic carbon was finally calculated from the amount of dichromate consumed by applying a correction factor of 1.33.

Lignin contents in sawdust, guinea corn husk and rice husk were determined using the detergent fiber method, particularly the acid detergent lignin (ADL) (Merck, Darmstadt, Germany) procedure [21]. The substrates were dried, ground and accurately weighed, and the samples were treated with an acid detergent solution to remove soluble materials, leaving the acid detergent fiber (ADF), which consists largely of cellulose and lignin. Thereafter, the remaining residue was filtered, washed and dried to constant weight, while the dried ADF residue was treated with 72% sulfuric acid, which hydrolyses and removes cellulose. The remaining insoluble residue was then washed, dried and weighed. The residue was then ashed in a muffle furnace. The difference between the dried residue and its ash represents lignin, while the difference between the contents of the ADF and lignin represents the cellulose content in percentage. The ADL was determined using Equation (2) below:

(2) $$ADL=\;\frac{Weight\;of\;dried\;residue-Weight\;of\;ash}{Weight\;of\;original\;sample}\;\times \;100$$

The nutrient mass balance analyses for the substrate inoculated with biofertilizer were determined at the different retention days of 5, 10 and 15 days using Equation (3) below.

Net nutrient concentration by mass = Final mass of nutrient after fermentation (M_F_) − Initial mass of nutrient before fermentation (M_I_) − mass nutrient of the inoculum (M_IN_) (3)

The nutrient mass at initial, final and inoculum was determined using Equations (4)–(6):

(4) $$M_{I}=\;\frac{C_{1}}{100}\times \;W_{I}$$

(5) $$M_{F}=\frac{C_{F}}{100}\times \;W_{F}$$

(6) $$M_{IN}=\frac{C_{IN}}{100}\times \;W_{IN}$$

where C_I_, C_F_ and C_IN_ are the concentrations at initial, final and inoculum, while W_I_, W_F_ and W_IN_ are the weights at initial, final and inoculum.

### 2.5. Experimental Design and Statistical Analysis

The experiment was arranged in a 4 × 3 factorial design consisting of three agricultural waste substrates and four contact periods with 9 independent replicates, with single measurement coming from different fermentation containers. Normality test was conducted on the substrate composition using the Ryan–Joiner approach, which is similar to the Shapiro–Wilk method (See Figure S1). The normality test was carried out in order to determine its suitability for further analyses (analysis of variance—ANOVA). A value of the Ryan–Joiner (RJ) statistics between 0.9 and 1 showed that data set are normally distributed (see Supplementary Data—Figure S1). Data obtained for total nitrogen, phosphorus, and potassium concentrations were subjected to analysis of variance (ANOVA—see ANOVA table in Tables S1–S3). Mean separation was performed using Tukey’s test at a significance level of *p* < 0.05. Statistical analyses were performed using Minitab software, version 18.0.

## 3. Results and Discussion

### 3.1. Chemical Composition of the Fungal Inoculum

Table 1 presents the chemical characterization of the fungal inoculum. The results indicate that the fungal inoculum exhibited high viability, with a propagule density standardized to 6.46 × 10^11^ CFU ml^−1^ (obtained with 10^−9^ dilution, 65 colonies and 0.1 plating), demonstrating its suitability for large-scale multiplication of *Trichoderma* sp.

High colony-forming unit (CFU) counts are indicative of active microbial growth and enhanced metabolic activity, which are essential characteristics of effective biofertilizers. Similar observations have been reported by Harman et al. [5], who noted that *Trichoderma* sp. possess rapid growth rates and strong colonization abilities that facilitate nutrient transformation and plant growth promotion. The nutrient composition reported in Table 1 further suggests that *Trichoderma* sp. effectively mineralized the lignocellulosic agricultural residues (decomposed litters of bamboo tree), thereby enhancing nutrient availability. The enhanced nutrient concentrations observed in the biofertilizer compared with previously reported values demonstrate the capacity of *Trichoderma* sp. to decompose complex organic substrates and release essential nutrients such as nitrogen (N), phosphorus (P), and potassium (K). This agrees with the findings of Mahato et al. [22], who reported that *Trichoderma* spp. improve nutrient availability through organic matter decomposition, nutrient solubilization, and stimulation of beneficial rhizosphere processes.

The high nutrient status of the biofertilizer is agronomically important because N, P, and K are the primary macronutrients required for crop growth and yield formation. Nitrogen supports vegetative development, phosphorus promotes root establishment and energy transfer, while potassium regulates enzymatic activity, osmotic balance, and stress tolerance. Therefore, the nutrient-rich nature of the produced biofertilizer indicates its potential to partially substitute inorganic fertilizers while simultaneously improving soil biological health.

### 3.2. Influence of Agricultural Waste Substrates and Contact Time on the Nutrient Composition of Trichoderma sp. Biofertilizer

The total organic carbon of the agricultural wastes, namely sawdust, rice husk and guinea corn husk, were 18.0 ± 1.05, 17.0 ± 1.02 and 45 ± 1.81%, while the cellulose contents were 42.8 ± 1.92, 30.7 ± 1.82 and 36.2 ± 1.85%, respectively. Also, the lignin contents were 26.3 ± 1.10, 16.0 ± 1.12 and 17.3 ± 1.12% for the sawdust, rice husk and guinea corn husk, respectively. The C:N ratios were 1452, 194 and 635 for sawdust, rice and guinea corn husk, respectively; Table 2 demonstrates that both substrate type and contact time significantly influenced the nutrient composition of the *Trichoderma* sp.-based biofertilizer. Distinct responses were observed for total nitrogen, phosphorus, and potassium among sawdust, rice husk, and guinea corn husk substrates. The nutrient composition at contact periods of 5, 10 and 15 days were higher than those obtained with the control for sawdust, guinea and rice husks. This implies that this technique can be successfully used to increase fertility in soil.

For the total nitrogen, a consistent increase in total nitrogen concentration was observed with increasing contact time across all substrates. Total nitrogen increased from 0.0124% to 0.17% in sawdust, from 0.0878% to 0.627% in rice husk, and from 0.0709% to 0.901% in guinea corn husk between days 5 and 15. The highest nitrogen concentration was recorded in guinea corn husk after 15 days (0.901%), whereas the lowest value was observed in sawdust after 5 days (0.0667%). These results suggest that guinea corn husk provided a more favorable nutritional and structural environment for fungal proliferation and nutrient accumulation. The increase in nitrogen with contact time may be attributed to the progressive decomposition of organic matter and microbial mineralization processes. *Trichoderma* sp. are known to secrete extracellular enzymes capable of degrading cellulose, hemicellulose, and other organic polymers, thereby enhancing nutrient release and availability [5,23]. The superior performance of guinea corn husk may also be linked to its relatively balanced carbon-to-nitrogen ratio and higher cellulose content, which support fungal growth and biomass production [24].

For the phosphorus, unlike nitrogen, phosphorus exhibited inconsistent temporal patterns among the substrates. Phosphorus concentrations fluctuated with contact time, with no statistically significant differences reported within substrates. Rice husk recorded the highest phosphorus concentration (2.497%) at day 15, whereas sawdust exhibited the lowest value (0.2403%) at day 10 during incubation. These fluctuations may be associated with dynamic phosphorus immobilization and mineralization processes occurring during fungal metabolism. The relatively high phosphorus content observed in rice husk may be explained by its ash fraction and inherent mineral composition. Rice-derived residues are known to contain appreciable amounts of phosphorus and silica, which can become increasingly available through microbial degradation [24].

For the potassium, its concentration increased significantly with contact time across all substrates. The highest potassium concentration (2.389%) was recorded in sawdust after 15 days, whereas the lowest value (1.467%) occurred in guinea corn husk after 5 days. The progressive increase in potassium likely reflects continued degradation of lignocellulosic materials and subsequent release of exchangeable potassium into the substrate matrix. The superior potassium accumulation observed in sawdust may be attributed to its structural characteristics, which provide an extensive surface area for fungal colonization and nutrient transformation. Previous studies have demonstrated that sawdust serves as an effective substrate for *Trichoderma* cultivation because of its high cellulose content and favorable physical properties that support fungal growth and enzyme production [25,26].

#### Agronomic Implications

The differential nutrient profiles observed among substrates indicate that agricultural waste selection can be tailored according to crop nutrient requirements. Guinea corn husk produced the highest nitrogen concentration and may therefore be more suitable for nitrogen-demanding crops such as maize. Although the *Trichoderma* sp.-derived biofertilizer substantially improves soil nutrient status and contributes to sustainable nutrient management [27], its nutrient concentrations are generally insufficient to completely replace conventional mineral fertilizers for high-yield commercial crop production. Instead, the biofertilizer could be best utilized as an organic nutrient amendment, a soil health enhancer, a microbial inoculant, and a complementary input within an Integrated Nutrient Management (INM) system. Its integration with mineral fertilizers could help achieve optimum crop productivity while improving long-term soil fertility and reducing environmental impacts. These synergistic soil and agronomic benefits have been previously reported by several researchers [27,28], when organic amendments are used together with mineral fertilizers.

Conversely, sawdust generated the highest potassium concentration, making it potentially valuable for crops requiring substantial potassium inputs during flowering and fruit development. Rice husk demonstrated superior phosphorus accumulation and could contribute to enhanced root growth and early crop establishment. Overall, the results demonstrate that agricultural waste type and incubation period are critical determinants of biofertilizer quality. The findings further support the concept of converting locally available agricultural residues into value-added biofertilizers, thereby promoting circular agriculture, waste valorization, and sustainable nutrient management.

### 3.3. Main and Interaction Effects of Contact Time and Agricultural Waste Substrates on Nutrient Accumulation

Table 3 presents the analysis of variance (ANOVA) examining the main and interaction effects of agricultural waste substrates and contact time on the concentrations of total nitrogen (TN), total phosphorus (P), and total potassium (K) in the *Trichoderma* sp.-based biofertilizer. The results revealed highly significant (*p* < 0.0001) effects of substrate type on all measured nutrients, indicating that the nutrient composition of the biofertilizer was strongly dependent on the characteristics of the agricultural waste used as the growth medium. Similarly, contact time significantly affected TN, P, and K concentrations, while the interaction between substrate type and contact time was significant for TN and total K and P. The details (F-value and *p*-value) of the analysis results can be found in the Supplementary Material (Tables S1–S3).

The supplementary analysis of variance (ANOVA) results demonstrated that both agricultural waste type (substrate) and contact time significantly influenced the nutrient composition of the *Trichoderma* sp.-based biofertilizer, although the magnitude of these effects varied among nitrogen (N), phosphorus (P), and potassium (K).

For total nitrogen, the analysis revealed that agricultural waste type exerted strong influence, suggesting that the inherent physicochemical properties of the substrates largely determined nitrogen enrichment during fungal fermentation. Contact time also significantly affected nitrogen accumulation (*p* < 0.001), confirming that prolonged incubation enhanced nitrogen (nutrient) mineralization through continued fungal activity and degradation of the lignocellulosic materials [29,30,31,32,33,34]. Furthermore, the significant interaction between substrate type and contact time (*p* < 0.001) demonstrated that nitrogen accumulation over time differed among the agricultural wastes. This indicates that the response of each substrate to fungal colonization and decomposition was substrate-dependent, reflecting differences in nutrient availability, degradability, and microbial utilization.

Similarly, the phosphorus analysis showed that both substrate type and contact time significantly influenced phosphorus concentration. Agricultural waste had a highly significant effect (*p* < 0.001), indicating that phosphorus availability varied considerably among the substrates, likely because of differences in their native phosphorus content and their susceptibility to microbial solubilization. Contact time also significantly affected phosphorus concentration (*p* < 0.001), suggesting that prolonged incubation enhanced phosphorus release through microbial production of phosphatases and organic acids. Moreover, like nitrogen, the interaction between substrate type and contact time was statistically significant (*p* < 0.0001). Therefore, substrate type and contact time synergistically influenced phosphorus content.

The potassium results also demonstrated significant effects of both agricultural waste type and contact time. Agricultural waste significantly influenced potassium concentration (*p* < 0.001), confirming that differences in substrate mineral composition affected potassium availability following fungal degradation. Contact time likewise had a significant effect (*p* < 0.001), indicating that continued incubation promoted the gradual release of potassium as decomposition progressed. In contrast to phosphorus, the interaction between substrate type and contact time was highly significant (*p* < 0.001), suggesting that potassium release patterns varied among substrates during fermentation. This interaction reflects differences in the structural composition and mineral-binding characteristics of the agricultural residues, which influenced the rate at which potassium became available during fungal degradation.

Collectively, these findings indicate that substrate type and incubation period both played a significant role in enhancing nutrient release, confirming that adequate fermentation time is essential for maximizing microbial mineralization. The significant interaction effects observed for the total nitrogen, phosphorus and potassium further demonstrate that nutrient dynamics during biofertilizer production depend on both substrate characteristics and incubation duration. These results underscore the importance of selecting suitable agricultural residues and optimizing contact time to improve nutrient recovery and enhance the quality of *Trichoderma* sp.-based biofertilizers for sustainable agricultural applications.

#### 3.3.1. Effect of Agricultural Waste on Nutrient Composition

The highly significant effect of agricultural waste on TN, P, and K demonstrates that substrate composition plays a fundamental role in determining biofertilizer quality. Agricultural residues differ considerably in their lignocellulosic composition, carbon-to-nitrogen ratio, mineral content, porosity, and degradability, all of which influence fungal colonization and nutrient transformation processes. The superior nutrient performance observed in guinea corn husk and rice husk substrates may be attributed to their favorable cellulose and hemicellulose contents, which provide readily degradable carbon sources that support fungal metabolism. *Trichoderma* sp. produce a wide range of extracellular enzymes, including cellulases, xylanases, and lignin-degrading enzymes, enabling efficient decomposition of agricultural residues and subsequent nutrient mineralization [5,7]. Enhanced decomposition increases the release of plant-available nutrients and contributes to the enrichment of the final biofertilizer product.

Furthermore, the significant substrate effect supports previous findings that agricultural waste selection is one of the most critical determinants of biofertilizer quality because different residues provide distinct nutrient reservoirs and microbial habitats [11]. Therefore, the choice of substrate should be guided not only by availability but also by its capacity to support microbial growth and nutrient enrichment.

#### 3.3.2. Effect of Contact Time on Nutrient Release

The highly significant influence of contact time on TN, P, and K concentrations indicates that nutrient accumulation in the biofertilizer is a dynamic process governed by fungal growth and substrate degradation. As incubation progressed, *Trichoderma* sp. likely increased its production of hydrolytic enzymes, leading to greater decomposition of complex organic compounds and enhanced nutrient release. During the early stages of incubation, nutrients remain largely bound within organic matrices. However, prolonged fungal activity accelerates mineralization and solubilization processes, increasing the availability of nitrogen, phosphorus, and potassium. Similar temporal increases in nutrient availability during fungal composting have been reported by Vinale et al. [35], who observed that prolonged colonization by *Trichoderma* enhances nutrient cycling and organic matter transformation. The significant contact time effect also suggests that insufficient incubation may limit nutrient enrichment, whereas extended incubation allows the fungal population to reach maximum metabolic activity. This observation highlights the importance of optimizing incubation periods to maximize biofertilizer quality and nutrient density.

#### 3.3.3. Interaction Between Agricultural Waste and Contact Time

The significant interaction effect between agricultural waste and contact time for TN and K indicates that the influence of incubation duration varied among substrates. This finding suggests that nutrient release patterns are not solely controlled by time or substrate independently but rather by the combined effects of both factors. The interaction reflects differences in decomposition rates among agricultural wastes. Substrates with lower lignin contents generally decompose more rapidly and release nutrients earlier, whereas highly lignified materials require longer periods for microbial degradation [36,37]. Rice husk is consistently described as lignocellulosic biomass with substantial lignin, with a value of 16.0%, which is within the range of 20 and 30% lignin [38]. Sawdust is likewise a woody lignocellulosic residue in which cellulose is tightly associated with lignin and hemicellulose, and wood-derived sawdust usually has lignin content ranging between 15 and 34% [39], which the value of 26.3% obtained in this study falls within. The guinea corn husk reportedly had lignin content between 14.6 and 20.7% [40], which the value of 17.3% obtained in this study falls within. Consequently, as a result of differences in their lignin contents, evident in the previous studies mentioned above, the response of TN and K to increasing contact time differs, which may be due to substrate characteristics. Such synergistic interactions have been widely reported in organic waste bioconversion systems where microbial decomposition depends on both substrate quality and incubation duration [10]. The presence of a significant interaction effect for phosphorus suggests that phosphorus concentration dynamics were influenced by both the substrate type and incubation time. Phosphorus release in organic substrates often depends on substrate mineral composition and phosphatase-mediated solubilization, including decomposition time [41].

#### 3.3.4. Implications for Biofertilizer Production

The results presented in Table 3 have important implications for sustainable biofertilizer production. First, they demonstrate that nutrient enrichment can be optimized through strategic selection of agricultural wastes. Second, they emphasize that incubation duration should be carefully managed to maximize nutrient release and microbial activity. Third, the significant interaction effects indicate that optimal incubation periods may vary according to substrate type, suggesting that a single standardized production protocol may not be appropriate for all agricultural residues. From an agricultural perspective, the findings support the conversion of locally available crop residues into nutrient-enriched biofertilizers, thereby reducing waste disposal problems while promoting circular bioeconomy practices. The ability of *Trichoderma* sp. to transform low-value agricultural wastes into biologically active fertilizers further highlights its potential as a sustainable alternative to synthetic fertilizers, contributing to improved soil fertility, enhanced microbial activity, and environmentally friendly crop production systems.

### 3.4. Nutrient Balance Analysis Based on the Concentration

Tables S4 and S5 (Supplementary Data) present the initial and final nutrient masses for the substrates (sawdust, guinea corn husk and rice husk), with a raw sample calculation also presented in the Supplementary Materials for sawdust after a 5-day incubation period. Table S5 shows considerable differences in the quantities of N, P and K remaining in the agricultural wastes after 5, 10 and 15 days of contact. For sawdust, the final nitrogen mass increased from 0.1551 g at 5 days to 0.1775 g at 10 days and 0.2274 g at 15 days. Similarly, phosphorus varied from 2.5250 g at 5 days to 0.5407 g at 10 days and subsequently increased to 1.9146 g at 15 days. Potassium remained relatively high throughout the treatment, with values of 5.0383, 4.9950 and 5.0766 g at 5, 10 and 15 days, respectively. The progressive increase in nitrogen mass in the sawdust treatment from day 5 to day 15 may indicate increasing nitrogen retention as biodegradation progressed due to the fungal activities. Similar observations in the decomposition by microbes and fungal substrates have been reported by some researchers [42,43,44]. For rice husk, Table 1 shows a marked increase in nitrogen from 0.9874 g at 5 days to 1.1484 g at 10 days and 1.2540 g at 15 days. Phosphorus also increased substantially from 3.8106 g at 5 days to 4.9940 g at 15 days, although the value at 10 days was lower (2.4266 g). Potassium showed comparatively little variation, with values of 4.0199, 3.9358 and 4.0440 g at 5, 10 and 15 days, respectively. The guinea corn husk treatment recorded the highest final nitrogen masses among the three materials, increasing from 1.5288 g at 5 days to 1.7402 g at 10 days and 1.8020 g at 15 days. Phosphorus decreased from 4.4704 g at 5 days to 2.5560 g at 15 days, while potassium increased progressively from 3.3374 g to 3.6660 g over the same period. These differences demonstrate that the three feedstocks behaved differently during treatment. Such variation is expected because the chemical composition of composting materials depends strongly on the characteristics of the original feedstock like cellulose and lignin contents, which influence the rate of decomposition [45,46].

#### Nutrient Concentration (By Mass) Balance Analysis for the Substrates (Sawdust, Rice Husk and Guinea Corn Husk) Inoculated with the Biofertilizer

Table 4 represents the difference between the final nutrient mass and the initial nutrient input and therefore provides the clearest indication of apparent change in nutrient mass calculated from the measured nutrient concentrations and the reported sample masses during the treatment period. For sawdust, nitrogen showed negative net balances at all three contact times: −0.2109 g at 5 days, −0.1885 g at 10 days and −0.1386 g at 15 days. In contrast, phosphorus and potassium showed positive calculated net balances throughout the treatment. At 5 days, the net phosphorus and potassium balances were 2.3590 and 4.8693 g, respectively; by 15 days they were 1.7486 and 4.9076 g. The negative nitrogen balance in sawdust suggests that, based on the reported concentration and sample-mass data, the calculated nitrogen mass in the final material was lower than the recorded initial nitrogen input. During aerobic controlled organic waste treatment, nitrogen may be transformed and potentially lost through ammonia volatilization [47]. However, these apparent changes should not be interpreted as definitive nutrient enrichment or depletion because changes in substrate dry mass during incubation can alter measured nutrient concentrations and calculated nutrient masses. Rice husk exhibited positive net balances for all three nutrients at all contact times. Nitrogen increased from 0.4331 g at 5 days to 0.6997 g at 15 days, while phosphorus increased from 3.6446 g to 4.8280 g. Potassium remained positive, increasing from 3.8099 g at 5 days to 3.8340 g at 15 days. These values indicate positive apparent nutrient balances based on the reported initial and final measurements; however, they do not by themselves demonstrate net nutrient accumulation on a fixed initial dry-substrate basis. Changes in substrate mass during incubation may contribute to the observed differences. Similar patterns were observed for the guinea corn husk. Guinea corn husk also showed positive net balances for all nutrients. Nitrogen increased from 1.0168 g at 5 days to 1.2900 g at 15 days. Phosphorus was 4.3104 g at 5 days and declined to 2.3960 g at 15 days, while potassium increased from 3.1974 g to 3.5260 g. The positive nitrogen balance is particularly notable because nitrogen is an essential nutrient required for microbial growth and is frequently subject to losses during organic waste treatment. Nevertheless, the observed positive nitrogen balance should be regarded as an apparent balance rather than definitive evidence of nitrogen retention or incorporation, because the present concentration-based assessment does not fully account for changes in total substrate dry mass during incubation.

Overall, the results indicate that rice husk and guinea corn husk demonstrated positive net balances for N, P and K, whereas sawdust demonstrated a negative nitrogen balance but positive phosphorus and potassium balances. The increasing nitrogen values observed for rice husk and guinea corn husk with increasing contact time may indicate progressive nutrient transformation during treatment, while the relatively stable potassium values suggest less pronounced changes in potassium mass, while phosphorus showed greater fluctuations, particularly in sawdust and guinea corn husk. This indicates that phosphorus behavior may have been influenced by biological transformation and changes in substrate mass. Therefore, the successful conversion of the substrates to biofertilizer would help convert waste to wealth and help ensure environmental sustainability, as documented in previous studies [48].

## 4. Conclusions

This study demonstrated the potential of *Trichoderma* sp. to bio-convert agricultural wastes into nutrient-enriched biofertilizers, thereby providing a sustainable approach for waste valorization and soil fertility enhancement. The results revealed that both agricultural waste substrate type and incubation period significantly influenced the nutrient composition of the produced biofertilizer. Total nitrogen, phosphorus, and potassium contents varied among substrates, indicating differences in nutrient release and microbial decomposition efficiency. Guinea corn husk produced the highest total nitrogen concentration (0.901%), rice husk yielded the highest phosphorus content (2.497%), while sawdust recorded the greatest potassium concentration (2.389%) after 15 days of incubation. The significant interaction between substrate type and incubation period further highlighted the importance of optimizing both factors to maximize nutrient enrichment.

Overall, the findings indicate that biofertilizers show nutrient enrichment under laboratory fermentation conditions, but field performance is required for validation. This technology offers a practical strategy for nutrient recycling, reduction in agricultural waste disposal problems, and promotion of sustainable crop production. Future studies should evaluate the agronomic performance of the produced biofertilizers under field conditions and assess their long-term effects on soil health, nutrient availability, and crop productivity.

## Figures and Tables

**Table 1:** Nutrient composition of the fungal inoculum from the decomposed litters of bamboo tree.

Parameter (%)	Value
Nitrogen	2.79 ± 0.1
Sodium	0.4595 ± 0.03
Total phosphorus	1.0275 ± 0.08
Total potassium	0.798 ± 0.07
Calcium	17.64 ± 1.5
Magnesium	10.20 ± 1.2
Manganese	0.036 ± 0.002
Iron	0.491 ± 0.03

Note: Data are presented as mean ± standard deviation.

**Table 2:** Effects of contact time and agricultural waste on the substrate-based biofertilizer nutrients.

Agricultural Waste	Contact Time (Days)	Total Nitrogen (%)	Phosphorus (%)	Potassium (%)
	control	0.0124 ± 0.001 ^c^	0.017 ± 0.001 ^c^	0.029 ± 0.001 ^c^
Sawdust	5	0.0667 ± 0.001 ^b^	1.086 ± 0.1 ^a^	2.167 ± 0.2 ^b^
	10	0.0789 ± 0.002 ^ab^	0.2403 ± 0.02 ^b^	2.22 ± 0.2 ^b^
	15	0.107 ± 0.03 ^a^	0.901 ± 0.08 ^a^	2.389 ± 0.3 ^a^
Rice husk	control	0.0878 ± 0.002 ^d^	0.04 ± 0.003 ^d^	0.045 ± 0.003 ^c^
	5	0.434 ± 0.02 ^c^	1.675 ± 0.2 ^b^	1.767 ± 0.3 ^b^
	10	0.522 ± 0.03 ^b^	1.103 ± 0.1 ^c^	1.789 ± 0.4 ^b^
	15	0.627 ± 0.03 ^a^	2.497 ± 0.5 ^a^	2.022 ± 0.4 ^a^
Guinea corn husk	control	0.0709 ± 0.002 ^d^	0.015 ± 0.001 ^b^	0.018 ± 0.002 ^a^
	5	0.672 ± 0.03 ^c^	1.965 ± 0.3 ^a^	1.467 ± 0.2 ^b^
	10	0.791 ± 0.04 ^b^	1.742 ± 0.3 ^a^	1.567 ± 0.3 ^b^
	15	0.901 ± 0.04 ^a^	1.278 ± 0.1 ^a^	1.833 ± 0.6 ^a^

Note: *Within each nutrient column, mean values followed by different superscript letters differ significantly according to Tukey’s test at p < 0.05. Comparisons were made among all substrate × incubation time treatment combinations.* Data are presented as mean ± standard deviation. Values of total nitrogen, phosphorus and potassium at control are for uninoculated substrate (day 0) and uninoculated substrates incubated at 5, 10 and 15 days, since they have approximately the same values, due to the short time of the incubation.

**Table 3:** Effects of agricultural waste type, incubation period, and their interaction on the nutrient composition of *Trichoderma* sp. biofertilizer.

		Total Nitrogen	Phosphorus	Potassium
Main effects				
	Agricultural waste (AW)	****	****	****
	Contact time (CT)	****	****	****
Interaction	AW × CT	****	****	****

Note: **** means significant at 0.0001.

**Table 4:** Nutrient mass balance for the inoculated substrates at different incubation times.

Agricultural Waste	Incubation Time (Days)	Total Nitrogen (g)	Phosphorus (g)	Potassium (g)
	5	−0.2109	2.3590	4.8693
Sawdust	10	−0.1885	0.3747	4.8260
	15	−0.1386	1.7486	4.9076
	5	0.4331	3.6446	3.8099
Rice husk	10	0.5941	2.2606	3.7258
	15	0.6997	4.8280	3.8340
	5	1.0168	4.3104	3.1974
Guinea corn husk	10	1.2282	3.6724	3.3074
	15	1.2900	2.3960	3.5260

## Data Availability

The data presented in this study are available on request from the corresponding author.

## Supplementary Materials

The following supporting information can be downloaded at: https://www.mdpi.com/article/10.3390/biology15191712/s1, Table S1: Analysis of Variance for Nitrogen Content; Table S2: Analysis of Variance for Phosphorus Content; Table S3: Analysis of Variance Potassium Content; Table S4: Initial Mass Nutrient; Table S5: Final mass nutrient; Figure S1: Normality test result for raw sawdust for the Total Nitrogen concentration.
