# Supplementary material for: Bioconversion of Agricultural Residues into Nutrient-Enriched Biofertilizers Using Trichoderma sp.: Effects of Substrate Type and Incubation Time
Source: Biology (Basel). 2026 Sep 26;15(19):1712. doi: 10.3390/biology15191712 (PMC13663401; doi:10.3390/biology15191712)
Supplement: Supplementary file 1 [file biology-15-01712-s001.zip › biology-4555091-supplementary.pdf]

Table S1: Analysis of Variance for Nitrogen Content

Source	DF	Adj SS	Adj MS	F-Value	P-Value
Model	15	10.6828	0.71219	331.95	0.0001
Linear	6	7.0865	1.18109	550.51	0.0001
Waste	3	5.4586	1.81952	848.08	0.0001
Contact time	3	1.6280	0.54266	252.93	0.0001
2-Way Interactions	9	1.5050	0.16722	77.94	0.0001
Waste*Contact time	9	1.5050	0.16722	77.94	0.0001
Error	92	0.1974	0.00215		
Total	107	10.8802			

Table S2: Analysis of Variance for Phosphorus Content

Source	DF	Adj SS	Adj MS	F-Value	P-Value
Model	15	78.1749	5.2117	672.02	0.0001
Linear	6	58.8077	9.8013	1263.83	0.0001
Waste	3	4.1564	1.3855	178.65	0.0001
Contact time	3	54.6514	18.2171	2349.02	0.0001
2-Way Interactions	9	1.5207	0.1690	21.79	0.0001
Waste*Contact time	9	1.5207	0.1690	21.79	0.0001
Error	92	0.7135	0.0078		
Total	107	78.8883			

Table S3: Analysis of Variance Potassium Content

Source	DF	Adj SS	Adj MS	F-Value	P-Value
Model	15	68.87	4.5911	25.12	0.0001
Linear	6	37.21	6.2014	33.93	0.0001
Waste	3	13.00	4.3339	23.71	0.0001
Contact time	3	24.21	8.0689	44.15	0.0001
2-Way Interactions	9	13.67	1.5190	8.31	0.0001
Waste*Contact time	9	13.67	1.5190	8.31	0.0001
Error	92	16.81	0.1828		
Total	107	85.68			

Table S4: Initial Mass Nutrient

Agricultural waste	Total input	Total nitrogen (g)	Phosphorus (g)	Potassium (g)
Saw dust		0.366	0.166	0.169
Rice husk		0.5543	0.22	0.21
Guinea corn husk		0.512	0.16	0.14

Table S5: Final mass nutrient

Agricultural waste	Contact time (days)	Total nitrogen (g)	Phosphorus (g)	Potassium (g)
	5	0.1551	2.5250	5.0383
Sawdust	10	0.1775	0.5407	4.9950
	15	0.2274	1.9146	5.0766
	5	0.9874	3.8106	4.0199
Rice husk	10	1.1484	2.4266	3.9358
	15	1.2540	4.9940	4.0440
	5	1.5288	4.4704	3.3374
Guinea corn husk	10	1.7402	3.8324	3.4474
	15	1.8020	2.5560	3.6660

Nutrient Mass Balance Calculation (Sample calculation for 5 days incubation time for sawdust)

1. Nitrogen in the inoculum

Inoculum mass = 12 g with a volume of 10 mL

Nitrogen content = 2.79%

$N_{inoculum} = \frac{2.79}{100} * 12 = 0.3348\ g\ N$

2. Nitrogen in the substrate before inoculation
   Substrate mass = 250 g
   Initial nitrogen content = 0.0124 %
   $N_{substrate} = \frac{0.0124}{100} * 250 = 0.0310\ g\ N$
   Therefore, total nitrogen entering:
   Nin = 0.366 k N
3. Nitrogen after 5 days
   Final mass = 232.5 kg
   Nitrogen content = 0.06675%
   $N_{out} = 232.5 * \frac{0.06675}{100} = 0.1552$ g N
4. Net nitrogen mass balance
   Net balance = $N_{out} - N_{in}$
   Net balance = 0.1552 – 0.3648

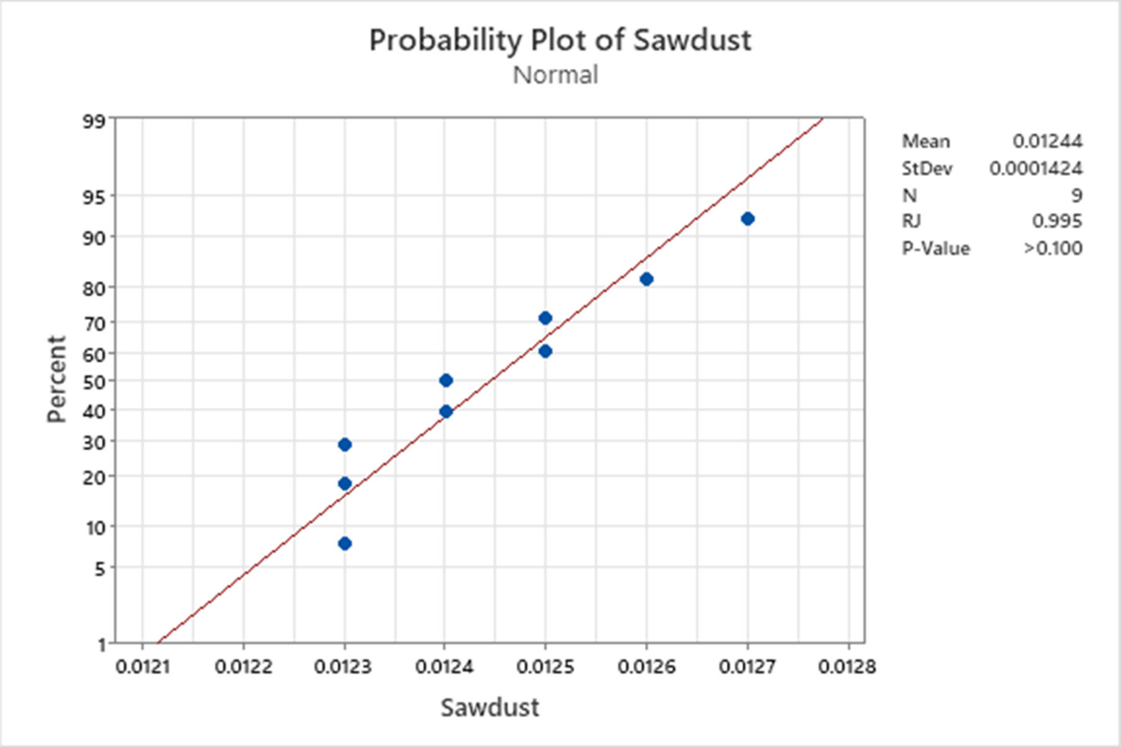

Figure S1: Normality test result for raw sawdust for the Total Nitrogen concentration
